# miR-28 modulates exhaustive differentiation of T cells through silencing programmed cell death-1 and regulating cytokine secretion

**DOI:** 10.18632/oncotarget.10731

**Published:** 2016-07-20

**Authors:** Qing Li, Nathan Johnston, Xiufen Zheng, Hongmei Wang, Xusheng Zhang, Dian Gao, Weiping Min

**Affiliations:** ^1^ Institute of Immunotherapy of Nanchang University, and Jiangxi Academy of Medical Sciences, Nanchang, China; ^2^ Department of Oncology, the Second Affiliated Hospital of Nanchang University, Nanchang, China; ^3^ Department of Surgery, Pathology and Oncology, Western University, London, Canada; ^4^ Lawson Health Research Institute, London, Canada

**Keywords:** exhausted T cells, miR-28, inhibitor receptors, PD1, melanoma

## Abstract

T cell exhaustion is a state of T cell dysfunction that arises during many cancer. miRNAs are one of major gene regulators which result in translational inhibition and/or mRNA degradation. We hypothesized that miRNAs exist that can silence PD1 and act as a modulator in vitro to revert exhaustive status of T cells. We demonstrated that the exhausted T cells with inhibitory receptors (IRs) are significantly increased in the melanoma-bearing mice. Meanwhile, the differentiated miRNA profiles in PD1+ exhaustive T cells were identified using a miRNA array; 11 miRNAs were observed with significant altered levels in the exhausted T cells isolated from melanoma-bearing mice. Among those identified miRNA candidates, miR-28 was capable of binding to multiple IRs based on an in silico analysis and subsequently silencing PD1, as demonstrated by a dual luciferase assay. Moreover, the expression of PD1 was attenuated after transfection with miR-28 mimic. The ability of miR-28 in regulating T cell exhaustion was further evidenced by the fact that the expression of PD1, TIM3 and BTLA of exhausted T cells was increased by the inhibitor of miR28. On the other hand, miR-28 also regulated the PD1+ Foxp3+ and TIM3+ Foxp3+ exhaustive Treg cells in vitro. miR-28 regulating T cell exhaustion was also observed by its ability in reinstalling impaired secretion of cytokines IL-2 and TNF-α by exhausted T cells. This study is the first to discover the effect of miR-28 on T cell exhaustion, providing novel targets with potential use as therapeutic markers in cancer immunotherapy.

## INTRODUCTION

Exhausted T cells were found in chronic viral infections and cancer, different inhibitory receptors were expressed increasing in those exhausted T cells with functional deficiency [1, 2]. Many inhibitory receptors such as programmed death-1 (PD1), T-cell immunoglobulin domain and mucin domain 3 (TIM3), B- and T-lymphocyte attenuator (BTLA), lymphocyte-activation gene 3 (LAG3), 2B4 (CD244), CD160, cytotoxic T lymphocyte antigen-4 (CTLA) are high-expressed on exhausted T cells during chronic infection [3, 4]. Dysfunctional tumor infiltrating T lymphocytes were also found to co-express PD1, LAG3 [5] and TIM3 [6]. PD1 is thought to be the most important cell surface inhibitory receptor among these molecules [7]. PD1 interacts with two ligands, programmed death ligand 1/2 (PD-L1/2) [8]. Interaction of PD1 with PD-L1/2 may negatively regulate cytokine secretion and T cells proliferation [7, 9].

Blocking the PD1 pathway is a new approach for treatment of tumor [10]. Studies on anti-PD1 antibodies (Nivolumab, Pembrolizumab, Pidilizumab and AMP-224) in various cancers including melanoma have entered clinical trials since late 2008 [11, 12]. Ultimately, research on blockade of the PD1 or PD-L1, holds much promise with greater response rates and lower toxicity due to the tumor-specific mode of activation rather than generalized suppression of T cell inhibition [13]. A review of the anti-PD1 and anti-PD-L1 antibodies currently in clinical trials shows encouraging response rates of between 20%-50% that have continued after treatment, and acceptable transient low-grade immune-related adverse effects [14]. Although PD1 antibodies are demonstrating significant benefits in melanoma treatment, no one has attempted to observe the effects of microRNA (miRNA) regulation in the PD1 pathway.

miRNAs are short (approximately 22 nucleotides) RNAs that regulate gene expression by binding to the 3’ untranslated regions (3’ UTR) of mRNAs, it will lead to translational inhibition or mRNA degradation [15]. miRNAs do not have perfect complementarity when in the double stranded stem loop or to the mRNA targets, which allows one miRNA to bind several mRNA transcripts. It is now believed that, in humans, over 1000 miRNAs regulate over 30% of our genome [16]. miRNAs originate from miRNA genes or introns of protein coding genes and, after processing by Drosha and Dicer, the miRNA is loaded into the RISC complex and oriented to the 3’ UTR of mRNAs, thus inducing repression of protein expression through mRNA degradation or translational inhibition [17, 18].

Overall, no experimental research has been done to observe the effects of miRNAs on PD1 in melanoma and T cell exhaustion. In this study we show valuable epigenetic data on the immunoinhibitory target PD1, which supports the use of miRNAs as prognostic markers and therapeutic molecules to confer T cell immunity against melanoma. We further describe miRNAs that can regulate PD1, and that in vitro transfection of miR-28 mimics acts therapeutically to reduce exhausted T cells and regulate the cytokine secretion in the tumor microenvironment.

## RESULTS

### Increased levels of exhaustion phenotype in T cells isolated from lymphoid and spleen in murine melanoma model

**Figure 1:**
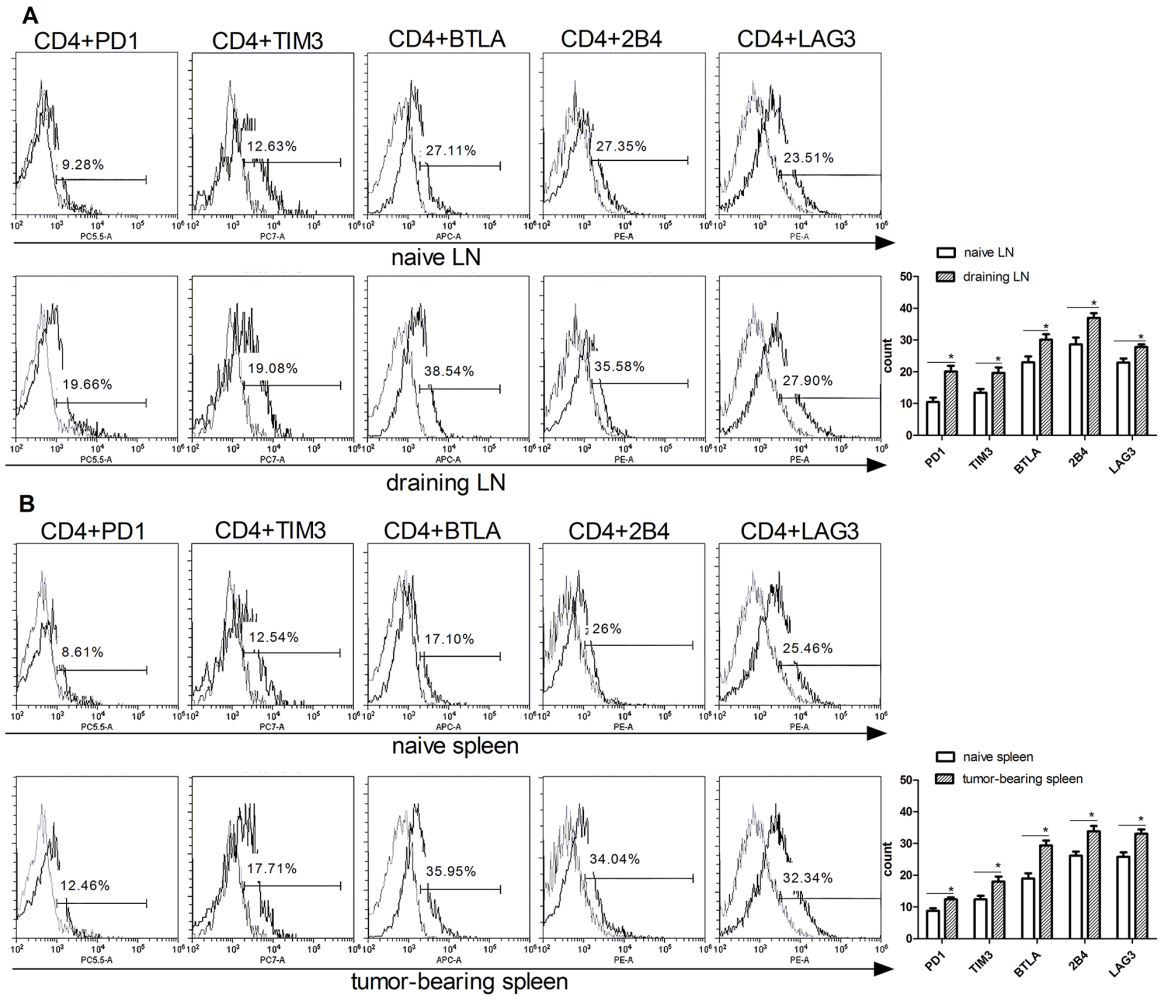
Upregulation of exhausted T cells phenotype in CD4 and CD8 T cells isolated from tumor-bearing mice T cells were isolated from the lymph nodes (naïve LN) and spleen (naïve spleen) of wild type mice, or draining lymph nodes (DLN), and spleen (T spleen) of B16F10-bearing mice. Exhausted T cell phenotypes (PD1, TIM3, BTLA, 2B4, LAG3) on CD4 **A.** and **B.** and CD8. **C.** and **D.** T cells were analyzed by flow cytometry. Statistic analysis of the expression of PD1, TIM3, BTLA, 2B4, LAG3 levels in CD4 (right panel in A and B) and CD8 (right panels in C and D) were performed from three independent experiments. Significance was assumed if the *P* value was less than 0.05 (*=*P*<0.05, **=*P*<0.01).

To confirm the T cell exhaustion in cancer, we first examined the expression of IRs such as PD1, TIM3, BTLA, 2B4 and LAG3 in T cells isolated from mice borne with B16F10 murine melanoma. C57BL/6 mice were inoculated subcutaneously with 4x10^5^ B16F10 cells on their backs, tumors were allowed to grow for 16 days before isolating lymphocytes from draining lymph nodes and spleen. Next, flow cytometer was used to detected the IRs levels (PD1, TIM3, BTLA, 2B4, LAG3) in T cell-containing tissues between naive and B16F10-bearing mice. PD1, TIM3, BTLA, 2B4, LAG3 were markedly increased in both CD4+ and CD8+ T cells of B16F10 tumor-bearing mice compared to naive mice (Figure 1). The CD4+PD1+ level was increased in draining lymph node (19%) than naïve mice (9%). CD4+TIM3+ was increased from 12% (naïve lymph nodes) to 19% (draining lymph node), other IRs phenotype such as CD4+BTLA+ (38%), CD4+2B4+ (35%), CD4+LAG3+ (27%) was also increased in draining lymph node of melanoma-bearing mice than naïve mice (Figure 1A). The exhausted phenotype of tumor bearing mice spleen were also detected. The similar growth was also found in the spleen T cells (Figure 1B). The IRs of CD8+ T cells were also have higher expression than in the naïve mice (Figure 1C–1D). These data suggested that the melanoma microenvironment has a significant impact on T cells exhaustion marker expression in the lymphoid and spleen tissue, the percentage of exhausted T cell was increasing when animals bear tumor.

### Differentiation of miRNA profiles between CD4+PD1- and CD4+PD1+ T cells isolated from tumor-bearing mice

**Figure 2:**
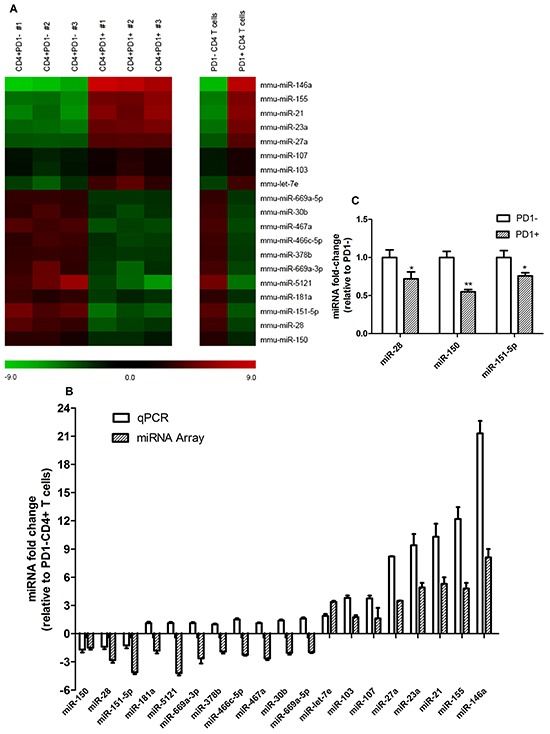
Global miRNA level profile between CD4+PD1- and CD4+PD1+ T cells isolated from tumor-bearing mice **A.** Using the Affymetrix GeneChip 3.0 miRNA Array, a total of 1,966 mouse miRNAs were analyzed in PD1+ and PD1- CD4+ T cells. The heat maps represent 19 miRNAs with a fold change greater than +/- 2.0 between CD4+PD1- and CD4+PD1+ samples. The left heat map shows fold changes between CD4+PD1- and CD4+PD1+ T cells from three individual experiments, and the right heat map shows the average fold-change. **B.** In order to discover discrepancies between the miRNA array and RT-qPCR data, fold-changes were compared and only the miRNAs with equal trends were considered for further study. **C.** Significantly down regulated miRNAs in PD1+CD4+ T cells. * = *P* < 0.05, ** = *P* < 0.01 and *** = *P* < 0.001. The data shown are representative of at least three independent experiments.

To analyze the miRNA expression profile between PD1+ and PD1- T cells, PD1+ and PD1- CD4+ T cells were sorted from lymph nodes and spleen of tumor-bearing mice using a FACS Aria III, and total RNA including miRNA was extracted. Using the Affymetrix GeneChip 3.0 miRNA array no significant differences were found between the miRNA level profiles of either tissue, so the data from the both tissues were pooled. A heatmap was generated looking at miRNAs with a fold change of +/- 2.0, which found 11 miRNAs significantly downregulated and another 8 significantly upregulated in CD4+PD1+ T cells in comparison with CD4+PD1- T cells (Figure 2A). In order to confirm the results of miRNA array, RT-qPCR analysis on these 19 miRNAs was performed. Figure2B displays discrepancies between the miRNA array and RT-qPCR data, showing that only 3 down-regulated miRNAs (miR-150, miR-28 and miR-151-5p) and 8 upregulated miRNAs (miR-let-7e, miR-103, miR-107, miR-27a, miR-23a, miR-21, miR-155 and miR-146a) showed similar trends in altered miRNA levels. miR-28, miR-150, and miR-151-5p levels in CD4+PD1+ T cells decreased by 30%, 45%, and 25%, respectively (Figure 2C). The results imply that the levels of at least 11 miRNAs are altered as either a cause or a consequence of increased PD1 in a B16F10-derived tumor.

### *In silico* analysis and a dual luciferase assay of miRNAs that may bind to the 3’ UTR of PD1

**Figure 3:**
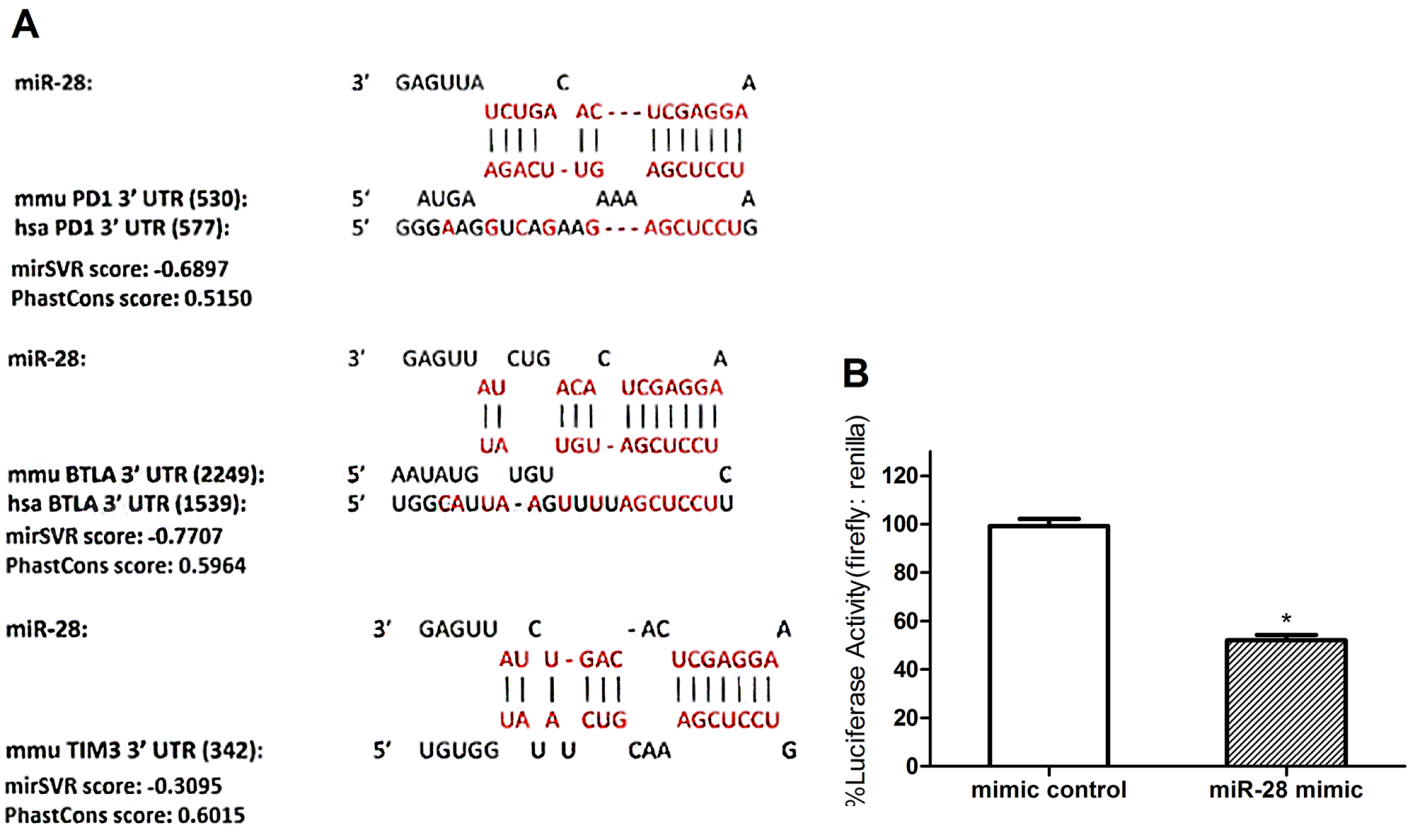
Defining the potential targets of exhaustion-associated inhibitory receptors PD1 by miR-28 **A.** *In silico* analysis using miRanda, TargetScan, PicTar and microRNA to discover miRNA candidates that may silence PD1, TIM3, and BTLA in various combinations. The theoretical bindings sites for miR-28 on the 3’ UTR of PD1, BTLA and TIM3. Each miRNA-mRNA combination displays the miRNA, murine 3’ UTR and human 3’ UTR sequences from top to bottom. The vertical lines represent base-pairing between the miRNA and the murine (mmu) 3’ UTR. The number in the bracket denotes the distance in nucleotides from the start of the 3’ UTR to the start of the miRNA seed region. All the mirSVR score < -0.1 and PhastCons score> 0.5. **B.** A Dual Luciferase Assay using pmirGLO Plasmid with PD1 3’ UTR insert and miRNA mimics. B16F10 cells were transfected with the PD1 3’ UTR dual luciferase plasmid and miR-28 mimic. Luciferase Activity was measured with a luminometer and normalized to mimic control. T test was used compared to the mimic control. Significance was assumed if *P*<0.05 (* = *P*< 0.05). The data shown are representative of three independent experiments.

To discover miRNAs that may bind to the 3’ UTR of PD1, TIM3, and BTLA, an *in silico* database search was conducted using miRanda, TargetScan, PicTar and microRNA (Figure 3). The sequences of all known conserved miRNAs were compared with that of the 3’ UTRs to discover areas of complementarity. Based on the base pairing in the seed region and other parts of the miRNA one can determine if a miRNA has the potential to bind to the 3’ UTR and prevent protein expression. Among the 11 miRNAs confirmed by RT-qPCR, miR-28 have significant complementarity to the 3’UTR of all 3 inhibitory immunoreceptor theoretically (Figure 3A). To determine whether miR-28 could silence PD1 through its 3’ UTR, a dual luciferase assay was conducted. The 3’ UTR of PD1 was amplified from wild-type C57BL/6 lymph node cells and inserted into the pmirGLO Dual Luciferase miRNA target expression vector directly downregulate of firefly luciferase [19]. B16F10 cells were used to transfect the dual luciferase plasmids with miR-28 mimic or control miRNA, cells were collected and analyzed for firefly and renilla luciferase activity 24 hrs later. miR-28 reduced luciferase activity by 50% (Figure 3B). These data indicate that miR-28 can reduce gene expression through the 3’ UTR of the PD1 gene. Therefore, in accordance with *in silico* and the dual luciferase assay, miR-28 was chosen as a candidate to determine if a miRNA can silence PD1 and regulate T cell function.

### Increased expression of inhibitory receptors in the in vitro-generated exhaustive T cell

**Figure 4:**
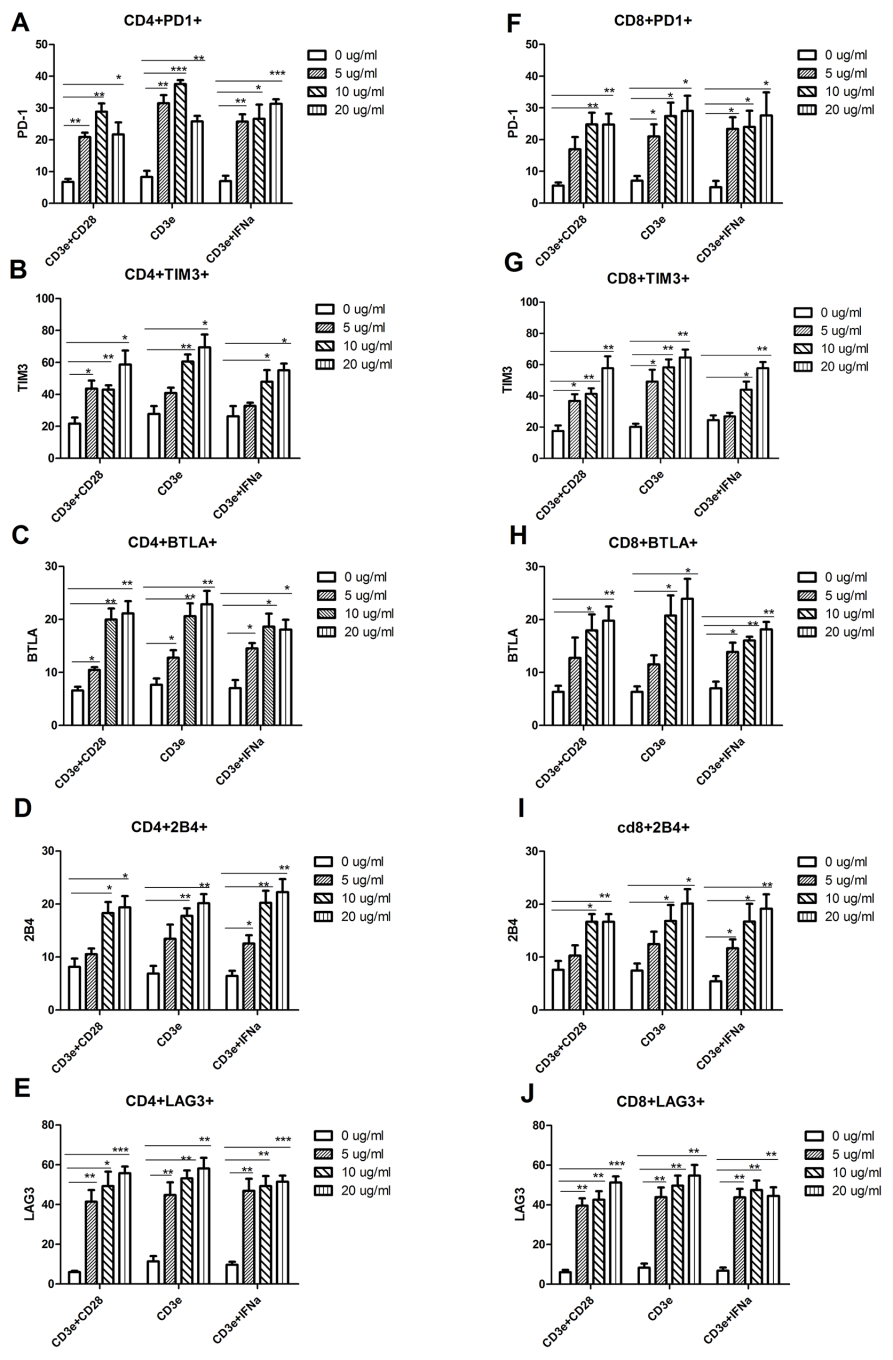
Increased expression of inhibitory receptors in the in vitro-generated exhaustive T cell Lymphocytes isolated from the lymph node of wild type C57BL/6 mice were cultured on anti-CD3e coating plates for 24 hrs, in the presence or absence of CD28 (1 μg/ml) or IFN-α (10 ng/ml). **A-E.** CD4+ T Cells were stained with PD1, TIM3, BTLA, 2B4 and LAG3 mAb, respectively, as described in Materials and Methods. **F-J.** CD8+ T Cells were stained with PD1, TIM3, BTLA, 2B4 and LAG3 mAb, respectively, as described above. The subsets of CD4+PD1 (A), CD4+TIM3 (B), CD4+BTLA (C), CD4+2B4 (D) and CD4+LAG3 (E) expression, as well as the subsets of CD8+PD1 (F), CD8+TIM3 (G), CD8+BTLA (H), CD8+2B4 (I) and CD8+LAG3 (J) were analyzed by flow cytometry. For all T cell subtypes, a one-way analysis of variance followed by a Dunnett's multiple comparison test was used to compare exhaustion phenotype between treatment and no treatment with CD3e, CD28 and IFN-α. Significance was assumed if the *P* value was less than 0.05 (*=*P*<0.05, **=*P*<0.01 and ***=*P*<0.001). The date shown are representative of three independent experiments.

Since the naturally low levels of PD1 on T cells from wild-type C57BL/6 lymphoid tissue makes it difficult to demonstrate miRNA-induced silencing, an *in vitro* system was needed that could upregulate inhibitory immunoreceptor levels. CD3e stimulation alone without CD28 co-activation signal causes the T cell to undergo anergy, a very similar process to T cell exhaustion. In addition, previous research has shown that IFN-α-stimulated cells in the tumor expressed high levels of PD1 [20]. Two methods were attempted in our research: culturing lymphocytes on anti-CD3e coated plates or anti-CD3e coated plates supplemented with IFN-α (anti-CD3e+IFN-α). 2x10^6^ lymphocytes were plated in each well of 24 well plates that were coated with 0, 1, 10, or 20 μg/ml of anti-CD3e overnight, with or without IFN-α (10 ng/ml) in cell culture medium, different concentrations of anti-CD3e (0, 1, 10, or 20 μg/ml) coating plate with an addition of CD28 co-activation as control. Cells were cultured for 24 hrs and analyzed by flow cytometry. Both Anti-CD3e and Anti-CD3e+ IFN-α treatment significantly increased exhaustion phenotype on CD4 (Figure 4A–4E) and CD8 T cells (Figure 4F–4J). There was no significant different between 10 μg/ml and 20 μg/ml group. Therefore, 10 μg/ml of anti-CD3e was used for subsequent experiments.

### miR-28 regulating the gene expression of PD1, TIM3 and BTLA

**Figure 5:**
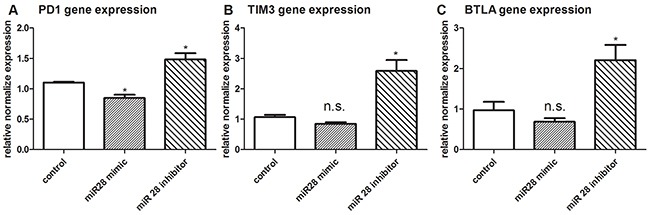
PD1, TIM3, BTLA gene expression after transfection with miR-28 mimic or miR-28 inhibitor Two million lymphocytes, collected from lymph nodes of B16F10-bearing mice, were transfected with 1 μg of miR-28 mimic or miR-28inhibitor, then cells were made exhaustive by the culture with anti-CD3e (10 μg/ml) coating plates for 72 hrs. The expression of PD1 **A.** TIM3 **B.** and BTLA **C.** were detected by qPCR. T test was used for the miRNA gene expression compared to the control group. Significance was assumed if the *P* value was less than 0.05 (* = *P*<0.05), n.s. (not significant) means *P* value was more than 0.05. The data shown are representative of at least three independent experiments.

To investigate the function of miR-28 to regulate the expression of IRs, T cells isolated from B16F10-bearing mice was transfected with miR-28 mimic or inhibitor. RT-qPCR was applied to test the gene expression of PD1, TIM3 and BTLA. The PD1 gene expression was decreased after transfection with miR-28 mimic (Figure 5A). On the contrary, the expression of PD1, TIM3 and BTLA were increased after transfection with miR-28 inhibitors (Figure 5A–5C). These data suggest that miR-28 is capable of regulating the PD1, TIM3 and BTLA genes on the T cell from melanoma-bearing mice.

### miR-28 manipulating exhaustive phenotype of T cells

**Figure 6:**
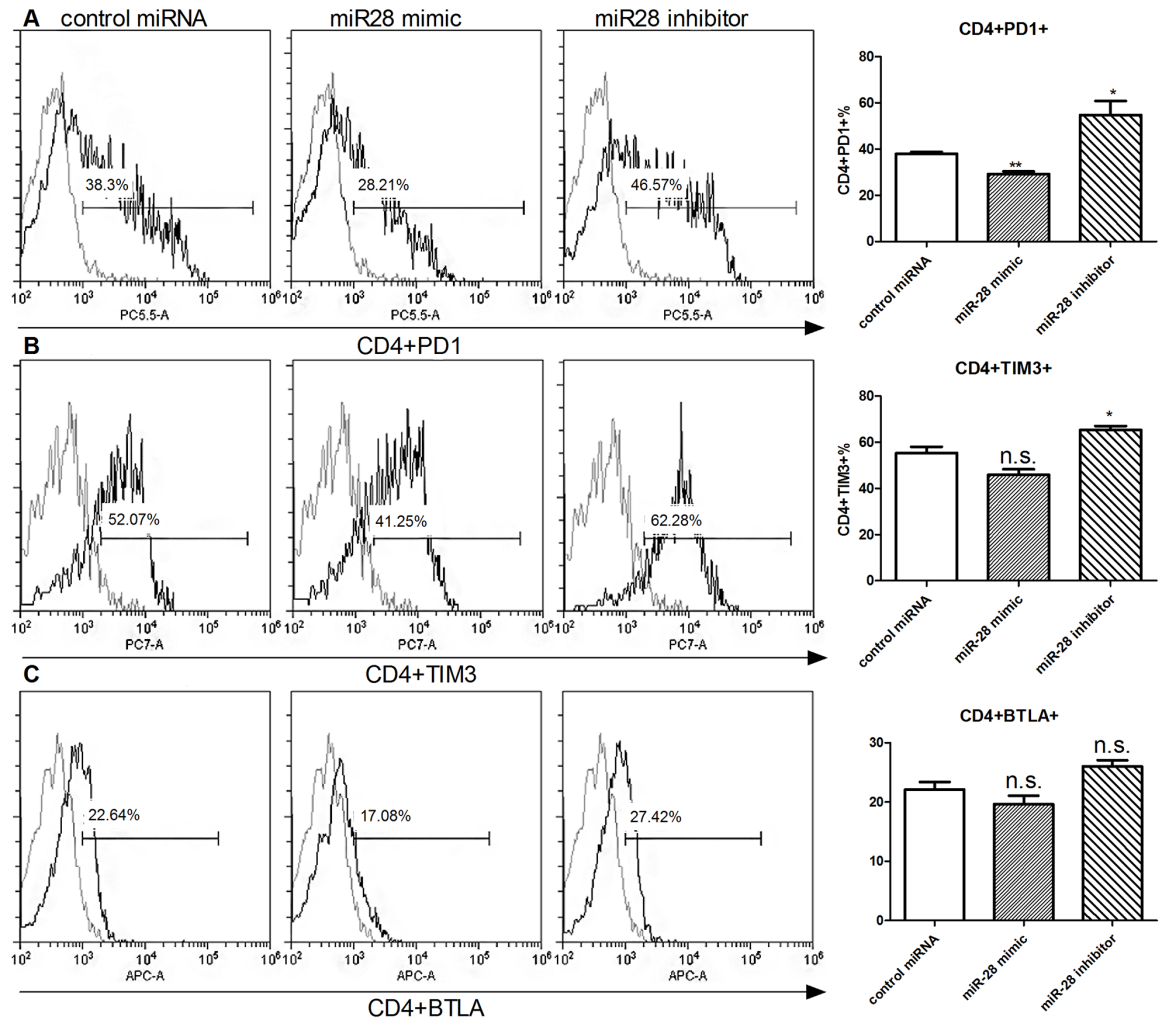
Alteration of exhaustive phenotype of T cells after transfection of miR-28 mimic and miR-28 inhibitor Two million lymphocytes, collected from lymph nodes of B16F10-bearing mice, were plated per well in a 24 well plate and transfected with 1 μg of miR-28 mimic or miR-28 inhibitor, then cells were cultured in anti-CD3e (10 μg/ml) coating plates for 72 hrs. Flow cytometric analysis were performed to determine the expression of exhaustion phenotype on CD4+ T cells **A-C.** and CD8+ T cells. **D-F.** Significance was assumed if *P*<0.05 using one-way analysis of variance followed by a Dunnett's multiple comparison test, and denoted as *=*P*<0.05 and **=*P*<0.01. n.s. (not significant) means *P* value was more than 0.05. The date shown are representative of three independent experiments.

Next, we investigated the function of miR-28 in regulating the exhausted phenotype. Flow cytometry was used to detect the exhaustion phenotype levels (PD1, TIM3, BTLA) in CD4+ or CD8+ T cell. After transfection with miR-28 mimic, PD1+ T cells were decreased from 38.3% to 28.21% in CD4+ T cells (Figure 6A) and from 36.91% to 28.5% in CD8+ T cells (Figure 6D), but TIM3 and BTLA had no significant decrease (Figure 6B, 6C, 6E, 6F). On the contrary, when transfected with miR-28 inhibitor, the PD1+ or TIM3+ T cells were increased both in the CD4+ (Figure 6A, 6B) and CD8+ T cells (Figure 6D, 6E). These results highlighted that miR-28 can convert the exhaustive phenotype of PD1+ T cells.

### miR-28 modulating FoxP3+PD1+ and Foxp3+TIM3+ Treg cells

**Figure 7:**
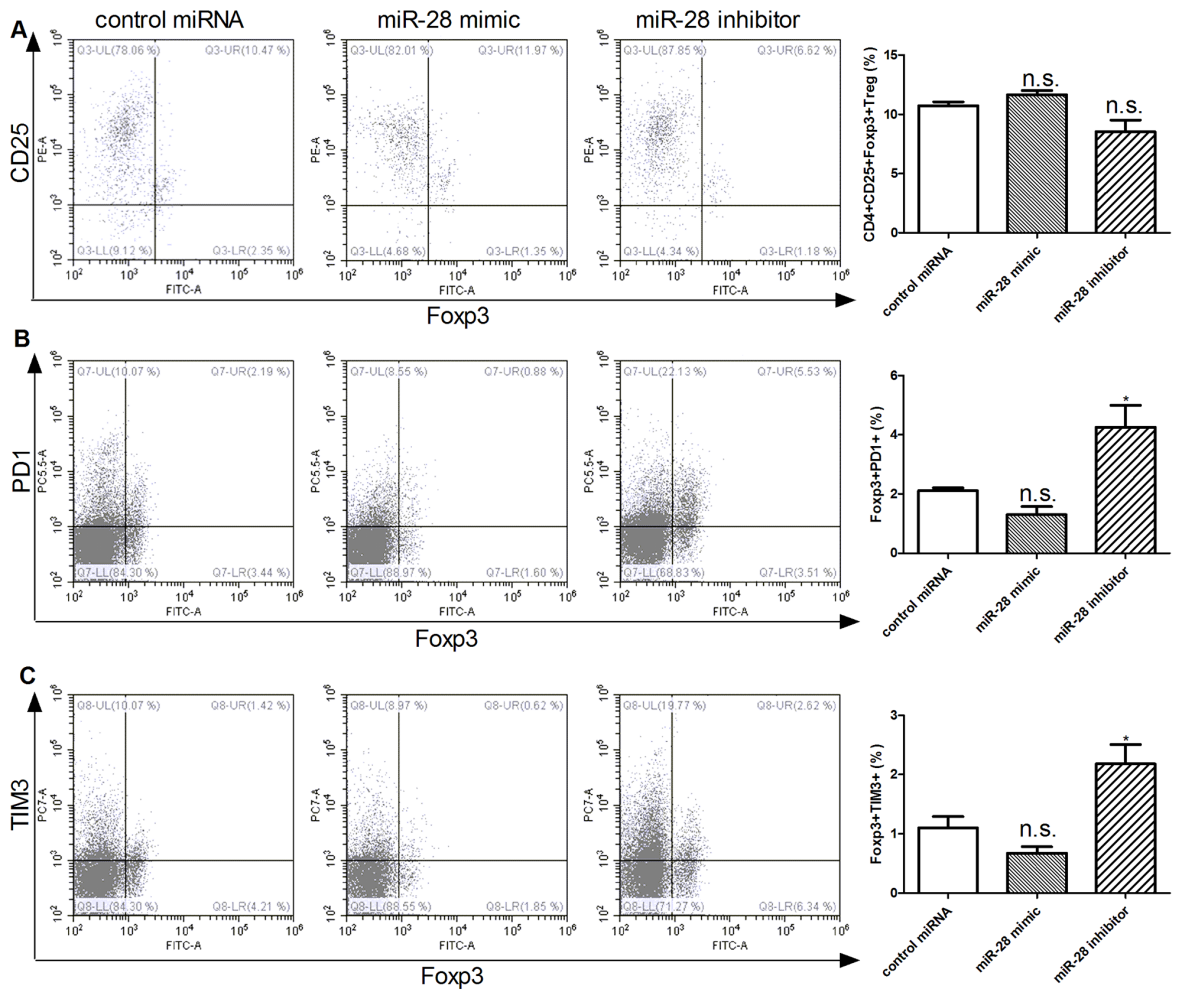
miR-28 regulating FoxP3+PD1+ and Foxp3+TIM3+ Treg cells Two million lymphocytes, collected from lymph nodes of B16F10-bearing mice, were plated per well in a 24 well plate and transfected with 1 μg of miR-28 mimic or miR-28 inhibitor, then cells were cultured with anti-CD3e (10 μg/ml) coating plates for 72 hrs. Flow cytometric analysis were performed to determine the subsets of conventional CD4+CD25+Foxp3+ Treg **A.** Foxp3+PD1+ T cells **B.** and **C.** Foxp3+TIM3+ T cells. The miRNA mimics or inhibitor group were compared to control group using an unpaired student's T test. Significance was assumed if the *P* value was less than 0.05 (* = *P*<0.05). n.s. (not significant) means *P* value was more than 0.05. The data shown are representative of at least three independent experiments.

It has been reported that Treg cells also undergo exhaustive differentiation, although it is controversial of the function of exhaustive Treg in tumor immunity [21]. It also remains largely unknown how exhaustive Treg cells are regulated. To clarify whether miR-28 is involved in the process of exhaustive differentiation of Treg cells, T cells isolated from spleen of B16F10-bearing mice, after transfected with miR-28 mimic or inhibitor in vitro with anti-CD3e coating plates, flow cytometry was used detected the conventional CD4+CD25+Foxp3+ Treg, as well as “exhaustive” Foxp3+PD1+ and Foxp3+TIM3+ Treg cells (Figure 7). The miR-28 mimic can decrease the expression of the Foxp3+PD1+ T cells. The Foxp3+PD1+ (Figure 7B) and Foxp3+TIM3+ cells (Figure 7C) were increased after transfected with miR-28 inhibitor. However, no significantly statistical difference was found in the conventional CD4+CD25+Foxp3+ Treg (Figure 7A) was found. Taken together, these data indicate that miR-28 may regulate PD1 and TIM3 expression in vitro partially.

### miR-28 recovering the cytokine secretion of exhausted T cells

**Figure 8:**
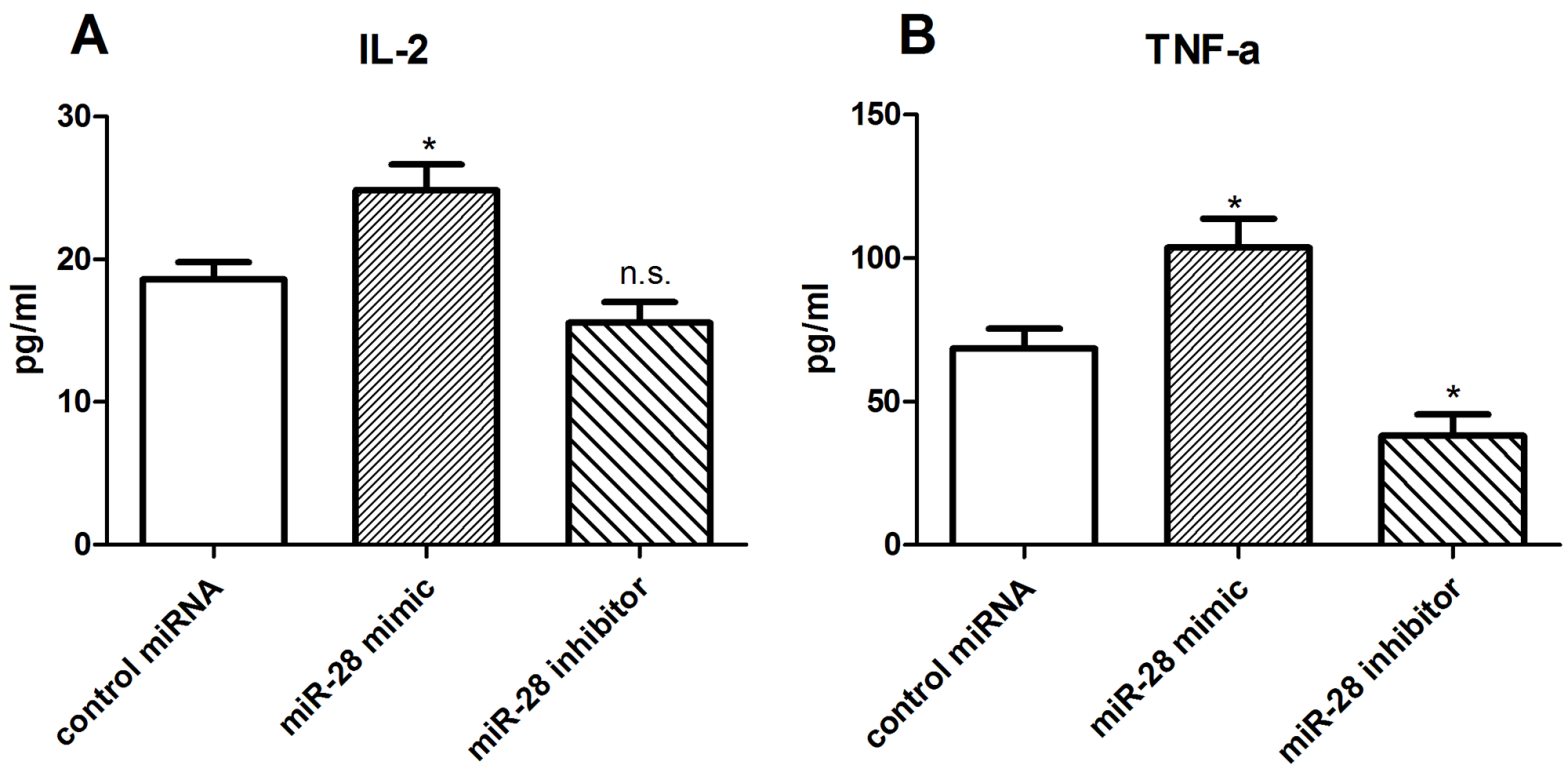
miR-28 modulating cytokine secretion of exhaustive T cells Two million lymphocytes, collected from lymph nodes of B16F10-bearing mice, were plated per well in a 24 well plate and transfected with 1 μg of miR-28 mimic or inhibitor, then cells were cultured with anti-CD3e (10 μg/ml) coating plates for 72 hrs. The supernatant of T cells were collected to detect the IL-2 **A.** and TNF-α **B.** using ELISA. Significance was assumed if *P*<0.05 by T test, and denoted as *=*P*<0.05, as comparing with control miRNA transfected cells. n.s. (not significant) means *P* value was more than 0.05. The date shown are representative of three independent experiments.

One of the characteristics of T cell exhaustion is impairing cytokine secretion by T cells [22]. To test whether miR-28 reverse the cytokine secretion of exhausted T cells, we collected T cells from the lymph nodes of B16F10-bearing mice, cultured in anti-CD3e coating plates, and transfected them with either miR-28 mimic or inhibitor. The supernatant of T cells were collected to detect cytokines including interleukin-2 (IL-2) and tumor necrosis factor-a (TNF-α) by enzyme-linked immune sorbent assay (ELISA). As shown in Figure 8, The concentration of IL-2 was increased from 18.62 pg/ml to 24.87 pg/ml when transfected with miR-28 mimic compared with control miRNA (Figure 8A), and the TNF-α level was increased from 68.52 pg/ml to 103.6 pg/ml. On the contrary, the miR-28 inhibitor decreased the TNF-α secretion (Figure 8B). Our results indicate that miR-28 may convert the exhaustive status of T cells through recover the ability of T cell to secret cytokines such as IL-2 and TNF-α.

## DISCUSSION

T cell exhaustion is a status of T cell functional deficiency in cancer [23]. The T cells progress through stages of dysfunction in tumor environment as the tumor antigen stimulate continuously [1], the dysfunctional T cells are fail to control the infection and the tumor cells. One of the main feature of exhausted T cells in cancer environment is overexpression of high levels of inhibitory receptors [4]. Researches indicated that exhausted CD8+ T cells up regulate multiple IRs, such as PD1, TIM3, BTLA [6, 24]. Those IRs correlate with variable levels of T cell dysfunction. In this paper, we have examined five IRs PD1, TIM3, BTLA, 2B4, LAG3 which are used to characterize exhausted T cells. We compared those exhausted T cell phenotype on CD4+ or CD8+ T cells between naive and melanoma-bearing mice. We found that all those IRs were increased in the CD4+ or CD8+ T cells in tumor-bearing mice. Higher expression of those IRs were found in T cells isolated from lymph node than from spleen.

In order to demonstrate how silencing of PD1 affects the function of T cells, PD1 was induced in vitro since naive T cells express very low levels of PD1. Anti-CD3e was chosen to up-regulate PD1 in T cells based on previous studies that demonstrated anti-CD3e is able to successfully to induce PD1 and T cell anergy on CD4^+^ T cells [25]. Previous research reported that IFN-α can stimulate T cells declined in number and became exhausted, and stimulated T cells in the tumor expressed higher levels of PD1 inhibitory receptor [20]. In our study, we optimized the protocol to induce exhausted T cells using anti-CD3e. We detected the effect of various concentrations of anti-CD3e antibodies and the effect of IFN-α. We found that both anti-CD3e and anti-CD3e+IFN-α can induce upregulation of inhibitory receptors in vitro. Not only PD1 were induced, the other IRs including TIM3, BTLA, 2B4 and LAG3 were increased as well. The concentration of anti-CD3e was positively correlated with the expression of those IRs within the range of 10 μg/ml and these was no remarkable difference in the expression of IRs between the concentrations of 10 μg/ml and 20 μg/ml. For cost-effectiveness, 10 μg/ml anti-CD3e Abs was chosen for in vitro experiments.

Until recently, PD-1 has been recognized as the primary Biomarker for exhausted T cells, PD1/PD-L1 pathway has a vital role to regulate immunosurveillance for tumors [26]. Blocking the PD-1/PD-L1 pathway are promising approaches for enhancing antitumor immune responses to accelerate tumor eradication [27]. Zhang et al found that miR-4717 can binding to the 3’UTR of PD1 mRNA and influence the PD-1 expression, it will affect the occurrence and progress of chronic HBV infection, and also change the immune states [28]. In non-small cell cancer, P53 can regulates PD-L1 via miR-34, which directly binds to the PD-L1 3’UTR [29]. In our research, the PD1+ and PD1- CD4 T cells was sorted for the microarray assays to compare miRNA expression between those two groups of cells. Based on our microarray and qPCR results, three miRNA (miR-28, miR-150, miR-151-5p) were confirmed decreased in CD4+PD1+ T cells. We postulated that the high expression of PD1 was associated with those three decreased miRNAs. Among the three miRNA, miR-28 has potential to bind to the 3’UTR of PD1, TIM3 and BTLA. An dual luciferase assay was conducted for PD1 3’UTR and demonstrated that miR-28 can directly silence PD1 through binding to its 3’UTR, melanoma cell line B16F10 were choose for the luciferase experiment, the cell line are easier to transfected and the results are more stabilized. So miR-28 was selected as the candidate miRNA that fulfilled the objectives of this study. miR-28 mimic silenced the 3’ UTR of PD1 and decreased PD1 expression. On the contrary, miR-28 inhibitors increased PD1, meanwhile, increased TIM3 and 2B4 expression.

Tumor infiltration of PD1 positive or Foxp3 positive lymphocytes, termed as exhaustive Treg cells, can used as significant prognostic indicators for clear cell renal cell carcinoma (CRCC). PD1 positivity and high number of Treg in a CRCC patient can predict poor overall survival of the patient [30]. Kaori Sakuishi et al also found that Foxp3+ Tregs in the tumor always express TIM3 and PD1. Those TIM3+ Tregs were activated in tumor environment, and have higher immunosuppressive activity [31]. The expression of the inhibitory molecules PD1 and LAG-3 was implicated in supporting Treg function [32, 33]. In this study, we found that the presence of miR-28 inhibitor can induce the Foxp3+PD1+ and Foxp3+TIM+ Treg cell differentiation in vitro. The results suggested that miR-28 can regulate the generation or differentiation of exhaustive Treg.

Changes in the cytokine production is a major feature of exhausted T cells in tumor microenvironment. IL-2, interferon (IFN-γ) and TNF-α are hyposecretion during T cell exhaustion. The production of IL-2, TNF-a and IFN-r secreted by PD1^hi^TIM3+ cells were reduced in leukemia patients [34]. In addition, Other cytokines such as IL-10 and TFG-β were also changed in T cells exhaustion [1, 22]. PD1 signaling may contribute significantly to the declination of IL2 and TNF-a production [8]. It was reported that TNF-α was a powerful anticancer cytokine to mediate cancer-related inflammation [35]. Liu et al reported that combined targeting of PD1 and TIM3 pathways increased the frequencies of TNF-α [36]. Blockage of the PD1/PD-L1 pathway in microglial can increase T-cell IFN-γ and IL-2 production [37]. Our research, for the first time, found that miR-28 can manipulating the secretion of cytokines from exhausted T cells. Addition of miR-28 mimics increase the level of IL-2 and TNF-α of T cells, suggesting another benefit of miR-28 that may restore the cytokine secretion function of exhausted T cells in tumor.

Most of researches were concerned about miR-28 and cancer cells in the past. In a non-immune cell related cancer, clear cell renal cell carcinoma, transfection with miR-28 mimic was shown to weaken mitotic checkpoint activation and to induce chromosomal instability by targeting MAD2L1 [38]. However, miR-28 was found high expression in other tumors such as esophagus cancer [39], ovarian cancer [40] and renal cancer [41]. miR-28 levels were reduced in B cell lymphoma and re-expression of this miRNA leads to impaired cell proliferation through silenced MAD2L1, a component of cell cycle for mitotic spindle coordination [42]. In our study, miR-28 was proved beneficial to inhibit T cells exhaustion. It is the first time to illuminate the relationship between miR-28 and T cells in melanoma.

Most of the clinical success of immunotherapy to treat the cancer is based on the checkpoint modulatory antibodies [43]. In 2014, the first anti-PD1 antibody pembrolizumab was approved for patients to treat advanced metastatic melanoma. There still have a lot of clinical trials to evaluate the antibodies targeting the inhibitory receptors such as CTLA4, PD1, PDL1 and LAGs for the treatment of patients with different cancer [44]. To our knowledge, there has been no reported research to restore exhausted T cells by using miRNA. Our study expands the role of miR-28 to that of an indirect tumor suppressor by decreasing the phenotype of exhaustion and regulating the cytokine IL-2 and TNF-α secretion.

## MATERIALS AND METHODS

### B16F10 cell culture

B16F10 cells were obtained from ATCC (Manassas, VA). Cells were cultured using DMEM medium (Gibco, Life Technologies, Burlington, ON) made complete with 10% fetal bovine serum (FBS; Gibco), 100 U/ml of penicillin (Gibco), and 100 μg/ml of streptomycin (Gibco) at 37 °C in 5% CO_2_.

### Melanoma mouse model

Eight-ten week old C57BL/6 mice (Charles River Canada, Saint-Constant, Canada) were used to generate a melanoma mouse model. Animals were housed under conventional conditions at the Animal Care Facility, Western University, and were cared for in accord with guidelines established by the Canadian Council on Animal Care. Tumors were generated on the backs of each mouse by subcutaneous injection of 4x10^5^ B16F10 cells in PBS (Gibco). Tumors were allowed to grow for 16 days or until reaching a size of 2,000 mm^3^. Mice were monitored daily and euthanized by CO_2_ inhalation.

### Lymphocyte isolation from lymph nodes and spleen

Draining lymph nodes and spleens were obtained from tumor-bearing mice, lymph nodes and spleens were also isolated from naïve mice. Lymph nodes were pressed through a 40 μm Falcon Cell Strainer (VWR, Mississauga, ON) into RPMI 1640 medium (Gibco) and cells were counted and centrifuged for 5 min at 1,500 rpm/4°C. Spleen cells were further isolated using ACK Lysing Buffer (Lonza) to lyse red blood cells.

### Cell sorting the CD4+PD1+ and CD4+PD1- cells

CD4+PD1^+^ and CD4+PD1^-^ T cells were isolated from tumor-bearing mice using fluorescence-activated cell sorting. Cell sorting was performed using a BD FACS Aria III (BD Biosciences, Mississauga, ON) after stained with 0.2 μg of CD8-FITC, CD4-PE, PD1-PerCP-eFluor 710, Rat IgG2b Isotype Control-PerCP-eFluor 710, or 0.1 μg of Fixable Viability Dye eFluor 506 (eBioscience).

### miRNA extraction and cDNA synthesis

Total RNA including miRNAs of sorting CD4+PD1+ and CD4+PD1- T cells was isolated using the miRNeasy Mini Kit (Qiagen, Toronto, ON) according to the manufacturer's instructions. RNA concentration was measured using a NanoDrop ND-1000 (Thermo Scientific, Ottawa, ON). RNA was reverse transcribed using the miScript II Reverse Transcriptase Kit (Qiagen).

### miRNA array analysis

An Affymetrix GeneChip 3.0 miRNA Array (Affymetrix, Santa Clara, CA) was used with the FlashTag Biotin HSR RNA Labeling Kit (Affymetrix) according to the manufacturer's instructions. The array was scanned using the GeneChip Scanner 3000 and Affymetrix GeneChip Command Console software (Affymetrix) and analysis of miRNA data was performed using the Partek Genomics Suite (Partek, St. Louis, MO). All miRNA expression data have been submitted to the Gene Expression Omnibus microarray data repository (http://www.ncbi.nlm.nih.gov/geo/query/acc.cgi?acc=GSE65208, accession number GSE65208).

### Reverse transcription qPCR (RT-qPCR) for assessment of miRNA level

RT-qPCR primers for those significantly altered miRNAs discovered from the miRNA array were purchased from Qiagen company (Qiagen). qPCR reactions were performed in a Stratagene Mx3000P QPCR System (Agilent Technologies, Lexington, MA) using the miScript SYBR green PCR Kit (Qiagen) and miScript Primer Assays (Qiagen). The PCR reaction conditions for miRNA analysis were 95° C for 15 min followed by 40 cycles of 94°C for 15 sec, 55°C for 30 sec, and 70°C for 30 sec. miRNA primer was referenced to homo sapiens small nucleolar RNA, C/D box 61 and normalized to PD1^-^ cells using the 2^-ΔΔCt^ method as described by Livak and Schmittgen [45].

### *In silico* analysis

*An in silico* analysis was performed by accessing multiple miRNA databases: miRanda, TargetScan Mouse 6.2 and PicTar. In each database, miRNAs that had complementarity to the 3’ UTRs of PD1, TIM3, and BTLA were searched. Only 7 mer or 8 mer miRNAs were considered.

### PD1 3’ UTR pmirGLO dual luciferase plasmid synthesis

The 3’ UTR of PD1 was amplified from wild-type c57BL/6 lymphocyte cDNA using PCR with 10 μM PD1 3’ UTR forward and reverse primer:

Forward: 5’-ATATACTCGAGCCAGATTCTTCAGCCATTAGCATGCT

Reverse: 5’-GCGTGTCTAGATTTAAAGCTTTTGGTACCATTTAATTATAACGGGCT and Taq polymerase (Invitrogen). The amplified cDNA was separated on a 1.5% agarose gel and the QIAquick Gel Extraction Kit (Qiagen) was used to isolate the PD1 3’ UTR cDNA. The PD1 3’ UTR and pmirGLO Dual Luciferase Plasmid (Promega, USA) were cleaved with XhoI and XbaI restriction enzymes (New England Biolabs, Ipswich, MA) and allowed to ligate overnight at 16° C. The PD1 3’ UTR pmirGLO Dual Luciferase Plasmid was amplified in JM109 cells (Promega, Madison, WI) and extracted using the GeneJET Plasmid Miniprep Kit (Fermentas, Burlington, ON). The concentration of the plasmid was then measured using a NanoDrop ND-1000. Sequencing for confirmation of successfully ligation was done using the BigDye Terminator v3.1 Cycle Sequencing Kit (Applied Biosystems, Foster City, CA) in an Applied Biosystems 3730 DNA Analyzer (Applied Biosystems).

### Dual luciferase assays and miRNA mimics

The miR-28 mimics sequence (Qiagen) transfected with the PD1 3’ UTR pmirGLO plasmid are as follows: 5’-AAGGAGCUCACAGUCUAUUGAG. AllStars Negative Control was used as miRNA mimic control (Qiagen #SI03650318). B16F10 cells were transfected using Lipofectamine 2000 according to the manufacturer's instructions using a 1:2 ratio of miRNA + plasmid (μg): Lipofectamine 2000 (μl). Cells were cultured in complete DMEM culture medium at 37°C and 5% CO_2_ for 24 hrs before analysis ina Lumat LB 9507 luminometer (Berthold Technologies, Oak Ridge, TN). The Dual-Luciferase Reporter Assay System (Promega) was used to detect firefly and renilla luciferase from the PD1 3’ UTR pmirGLO plasmid. Luciferase activity (firefly luciferase/renilla luciferase) was normalized to the miRNA mimic control and PD1 3’ UTR pmirGLO transfection.

### Lymphocytes treatment with anti-CD3e, CD3e+IFNa, CD3e+CD28

Lymphocytes isolated from lymph nodes of C57BL/6 mice were treated with anti-CD3e (BD Biosciences) or anti-CD3e+IFN-α (Pbl Assay Science, USA) or anti-CD3e+CD28 by plating 2x10^6^ cells in each well in 24 well plates. The plates were coated with various concentrations (from 0 – 20 μg/ml) of anti-CD3e in 200 μl of PBS overnight at 4° C. Plates were washed with PBS before lymphocytes were plated in Complete RPMI 1640. IFN-α (10 ng/ml) or CD28 (1 μg/ml) was added to wells as indicated in results section at the time of plating. With all treatments, lymphocytes were incubated at 37 °C and 5% CO_2_ for 24 hrs before cells were collected for flow cytometry.

### T cell transfection with miR-28 mimics and inhibitors

Lymphocytes from the lymph nodes of B16F10-bearing C57BL/6 mice were plated 2 million per well in a 24 well plate using 400 μl of RPMI 1640 with 10% FBS and 50 mM 2-ME (Transfection RPMI 1640 medium). One μg of miR-28 mimic/miR-28 inhibitor (controls, Qiagen #SI03650318 and #1027271) was added to 50 μl of Opti-MEM. Lipofectamine 2000 was added to separate tubes with 50 μl of Opti-MEM at a 2:1 ratio of miRNA(μg): Lipofectamine 2000 (μl). After 5 min incubation at room temperature, the contents of RNA and transfection reagent tubes were combined and incubated for 20 min at room temperature. The 100 μl mixture was then added to each well. After 4 hrs incubation at 37°C and 5% CO_2_, an additional 500 μl of RPMI 1640 medium containing 10% FBS and 50 mM 2-ME was added to each well and the plates were incubated for up to 72 hrs at 37°C and 5% CO_2_ before cells were collected for various experiments.

### PD1, TIM3 and BTLA gene expression

cDNA was synthesized using oligo-(dT) primer and reverse transcriptase (Invitrogen) according to the manufacture's protocol. The primers for PD1, TIM3 and BTLA and GAPDH were synthesized by Invitrogen, the sequences of oligonucleotide primers were shown in Supplementary Table S1. qPCR reactions were conducted using the SensiFAST^TM^ SYBR NO-ROX Kit (Bioline, USA). The PCR reaction conditions were 95° C for 2 min followed by 40 cycles of 95° C for 10 sec, 58°C for 10 sec, and 72° C for 20 sec, then 95° C for 10 sec. Results were converted from cycle threshold (Ct) values to transcript quantities follow the 2^-ΔΔCt^ method with mouse glyceraldehyde-3-phosphate dehydrogenase (GAPDH) as the endogenous control for target gene expression. The levels of PD1, TIM3 and BTLA after transfected with miR-28 mimic or inhibitor were then expressed relative to that control miRNA group.

### Flow cytometry

Flow cytometry was performed using a CytoFLEX S (Beckman Coulter Life Sciences, Mississauga, ON). Lymphocytes were stained with 0.2 μg of CD4-FITC, CD8-FITC, PD1-PerCP-eFluor 710, TIM3-PE-CY7, BTLA-APC, 2B4-PE, LAG3-PE, Rat IgG2b Isotype Control-PerCP-eFluor 710, Mouse IgG2a K Isotype Control-PE, Mouse IgG1 K Isotype Control-APC, CD4-PE-CY5, CD25-PE, Foxp3-FITC (eBioscience, San Diego, CA), or 0.1 μg of Fixable Viability Dye eFluor 506 (eBioscience)based on the different experiment.

### Statistical analysis

A two-tailed unpaired Student's T test was used to determine significance between two groups and a one-way analysis of variance for experiments with three or more groups. Details of control groups can be found in each figure legend. Significance was assumed at *P* values < 0.05.

## SUPPLEMENTARY MATERIALS TABLE



## Abbreviations

miRNA: microRNA
PD1: programmed death-1
TIM3: T-cell immunoglobulin domain and mucin domain 3
BTLA: B- and T-lymphocyte attenuator
IRs: inhibitory receptors
Treg: regulatory T cells
3’UTR: 3’ untranslated regions.
