# Supplementary material for: miR-28 modulates exhaustive differentiation of T cells through silencing programmed cell death-1 and regulating cytokine secretion
Source: Oncotarget. 2016 Jul 20;7(33):53735–50. doi: 10.18632/oncotarget.10731 (PMC5288217; doi:10.18632/oncotarget.10731)
Supplement: Supplementary file 1 [file oncotarget-07-53735-s001.pdf]

miR-28 modulates exhaustive differentiation of T cells through silencing programmed cell death-1 and regulating cytokine secretion

SUPPLEMENTARY TABLE

Supplementary Table S1: The sequences of oligonucleotide primers for qPCR

Target gene	Forward sense (5′→3′)	Reverse sense (5′→3′)
GAPDH	TGATGACATCAAGAAGGTGGTGAA	TGGGATGGAAATTGTGAGGGAGAT
PD1	CCGCTTCCAGATCATACAG	CTCTGGCCTCTGACATACTTG
TIM3	AGTGGGAGTCTCTGCTGGGTTGA	AGGATGGCTGCTGGCTGTTGA
BTLA	TGCAGGAGCCAGAAGAGAAAGTCA	CAATGTGGGGGTCAGGGATGG
